# Fractal interwoven resonator based penta-band metamaterial absorbers for THz sensing and imaging

**DOI:** 10.1038/s41598-022-23390-8

**Published:** 2022-11-17

**Authors:** Hurrem Ozpinar, Sinan Aksimsek

**Affiliations:** 1 Department of Electronics and Communications Engineering, Yildiz Technical University, Esenler, 34220 Istanbul Turkey; 2 ASELSAN Inc., 06200, Ankara, Turkey

**Keywords:** Engineering, Sensors and biosensors, Metamaterials

## Abstract

This paper presents a unique penta-band metamaterial absorber platform for terahertz imaging systems.The proposed fractal metamaterial absorber (FMMA) consists of fractal triangle section metasurfaces. By combining fractal resonators posing different operation skills in the same unit cell, the absorber shows multiband spectral response. The proposed unit cell structure operates at five near perfect absorption modes corresponding to the frequency bands of 1.1 THz, 3.4 THz, 4.9 THz, 5.9 THz, and 7.8 THz, respectively. Based on the fractal metamaterial absorber array, we also propose a sensing pixel design for bimaterial cantilever array sensing systems. The single pixel assembles 4*x*6 fractal resonator array and $SiO_2$-*Al* bimaterial microcantilevers. The sensing region of the FMMA pixel can bend the bimaterial cantilevers effectively at multiple modes, enhancing the imaging capacity. The effective medium theory is executed to visualize the constitute parameters during the absorption and reveal the origin of the rising modes. The absorption mechanism is also discussed based on the surface current distributions and electric field profiles. The numerical outcomes prove that the proposed fractal metamaterial unit cell is a promising candidate as an absorbing platform for THz band sensing and imaging applications. The derived iterative formula used in the fractal design procedure is explained for further investigations of microelectromechanical systems (MEMS) compatible compact absorber arrays.

## Introduction

In recent years, metamaterials have gained significant attention in both science and engineering due to their exotic electromagnetic properties which do not exist in nature^1^. Metamaterials, also called artificially engineered materials, pose an excellent ability to manipulate the incident electromagnetic radiation at sub-wavelength dimensions, leading state of art technologies such as ultra-wide band communications^2^, super lensing^3^, thermal radiation^4^, medical imaging^5^, polarization conversion^6^ and perfect absorption^7^. In particular, extensive studies on perfect absorber applications of the metamaterials have pushed the limits of the current systems, from microwaves to visible regimes. The first perfect metamaterial absorber was reported by Landy et. al. in 2008^8^, investigating an electric resonator-based absorber for X-band applications. Then many absorbing platforms have been reported for various applications involving radar detection^9^, solar energy harvesting^10^, bolometer^11^, stealth technology^12^, sensing, and imaging^13^. Particularly, absorbent metamaterials compatible with micro-electro-mechanical systems (MEMS), or microsystems-based fabrication have been at the center of special interest for THz band sensing/imaging technologies^11–13^. The metamaterial unit cell dimensions required for THz interaction are of the order of tens of micrometers, and micro-fabrication processes based on photolithography can provide high precision^14^.

**Figure 1:**
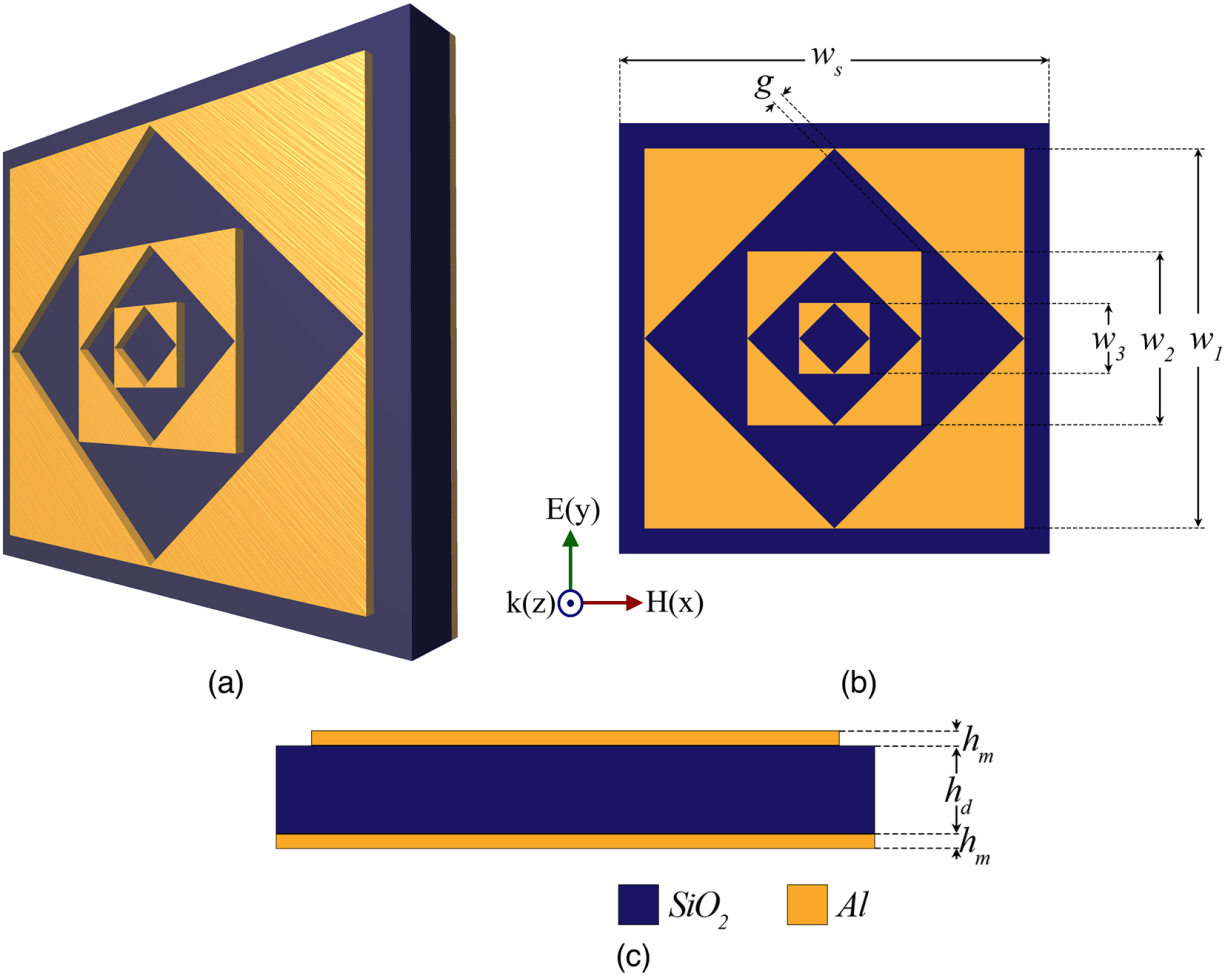
The configuration of fractal metamaterial absorber (FMMA) (**a**) 3D-view, (**b**) Top view with dimensions (**c**) Side view. The figure was created using Adobe Photoshop CC 2022 (Version 23.4.1, https://www.adobe.com/products/photoshop).

**Figure 2:**
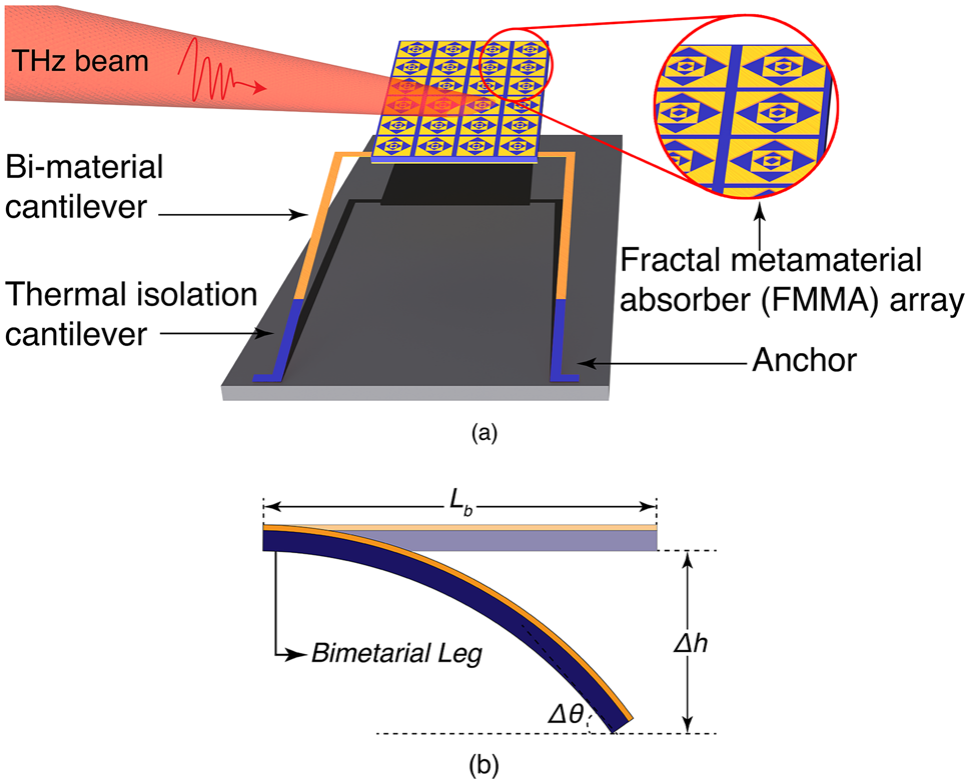
FMMA based sensing pixel (**a**) 3D view. (**b**) Illustration of thermomechanical deflection of the bimaterial sensor. The figure was created using Adobe Photoshop CC 2022 (Version 23.4.1, https://www.adobe.com/products/photoshop).

**Figure 3:**
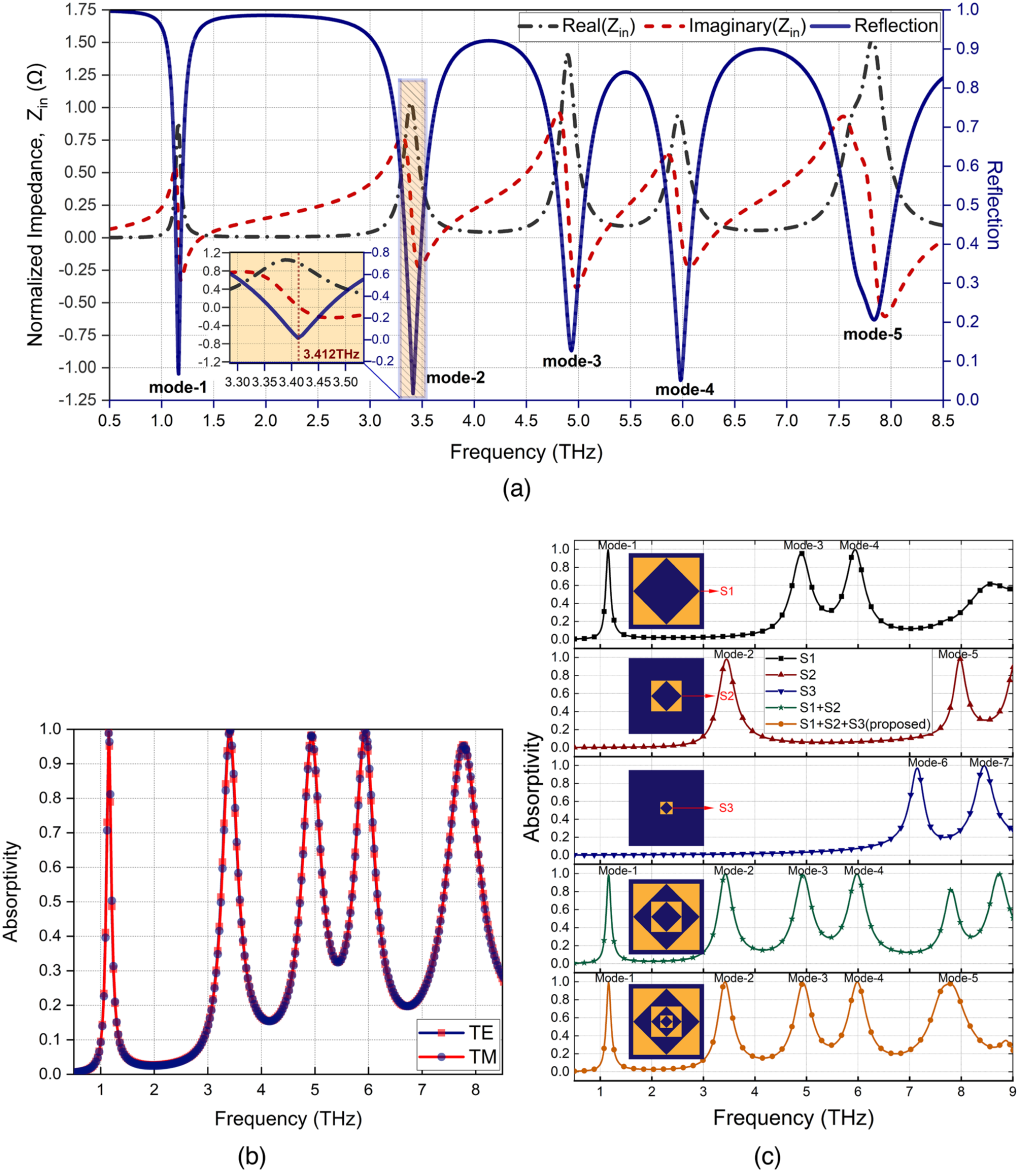
(**a**) Calculated normalized input impedance of the proposed absorber. (**b**) Absorption spectra at the normal incidence for TE and TM polarizations. (**c**) Absorptivity of each resonator and final design.

**Figure 4:**
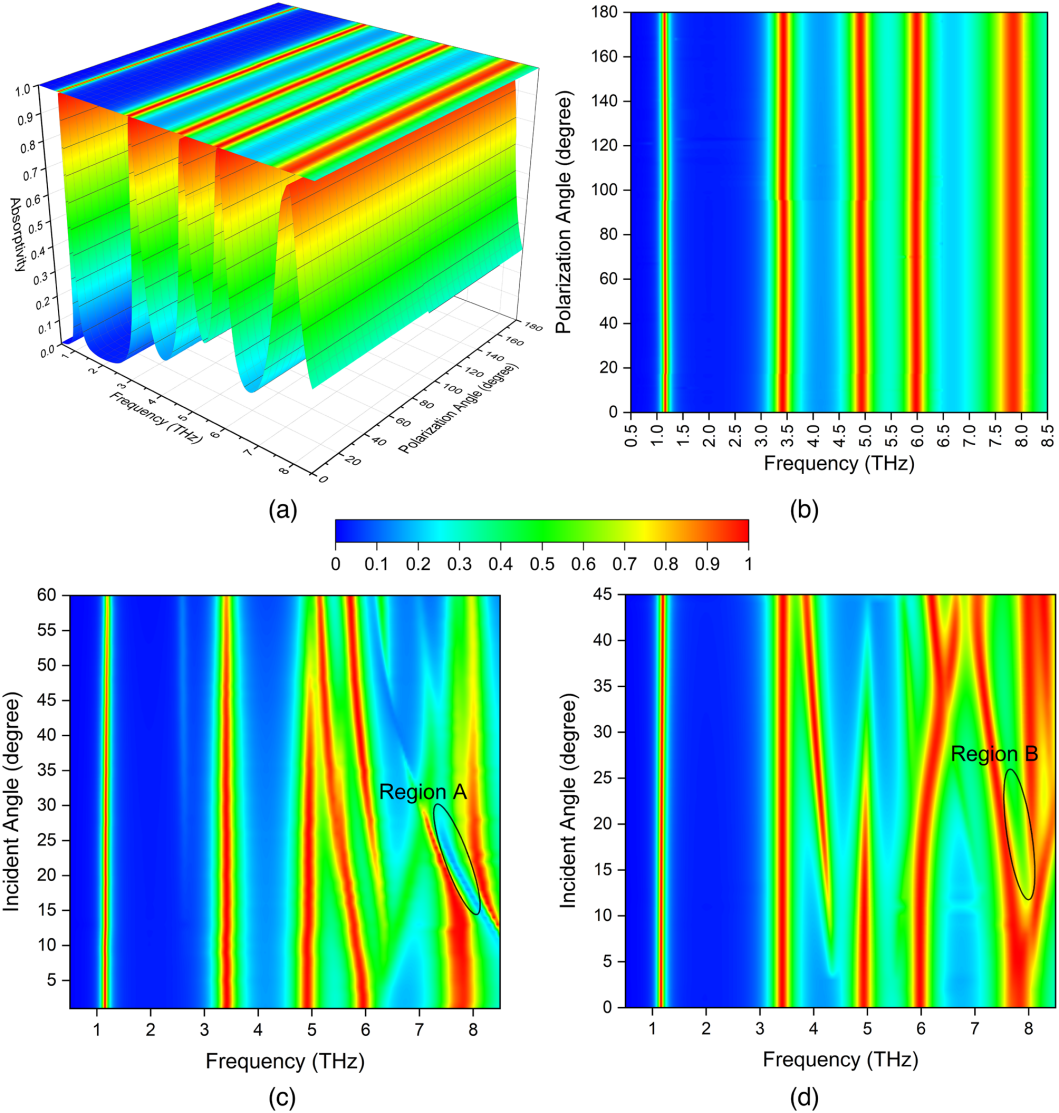
(**a**) 3-D contour plot of the absorption spectra of the proposed metamaterial absorber for polarization angle ($\phi$) ranging from $0^\circ$ to $180^\circ$ with $1^\circ$sweeping for normal incidence. (**b**) 2-D contour plot of the absorption spectra. (**c**) 2-D contour plots of the absorption spectra upon the different incident angles ($\theta$) for TE and (**d**) TM polarization.

**Figure 5:**
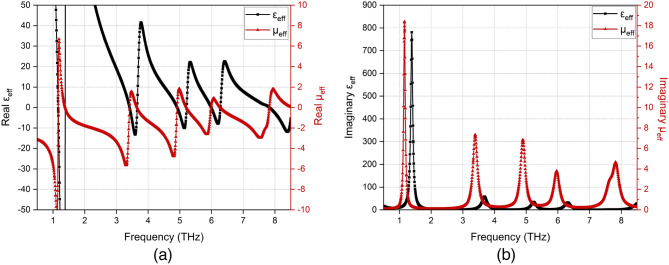
Calculated effective medium parameters: (**a**) Real parts of $\epsilon _{eff}$ and $\mu _{eff}$ (**b**) Imaginary parts of $\epsilon _{eff}$ and $\mu _{eff}$.

**Figure 6:**
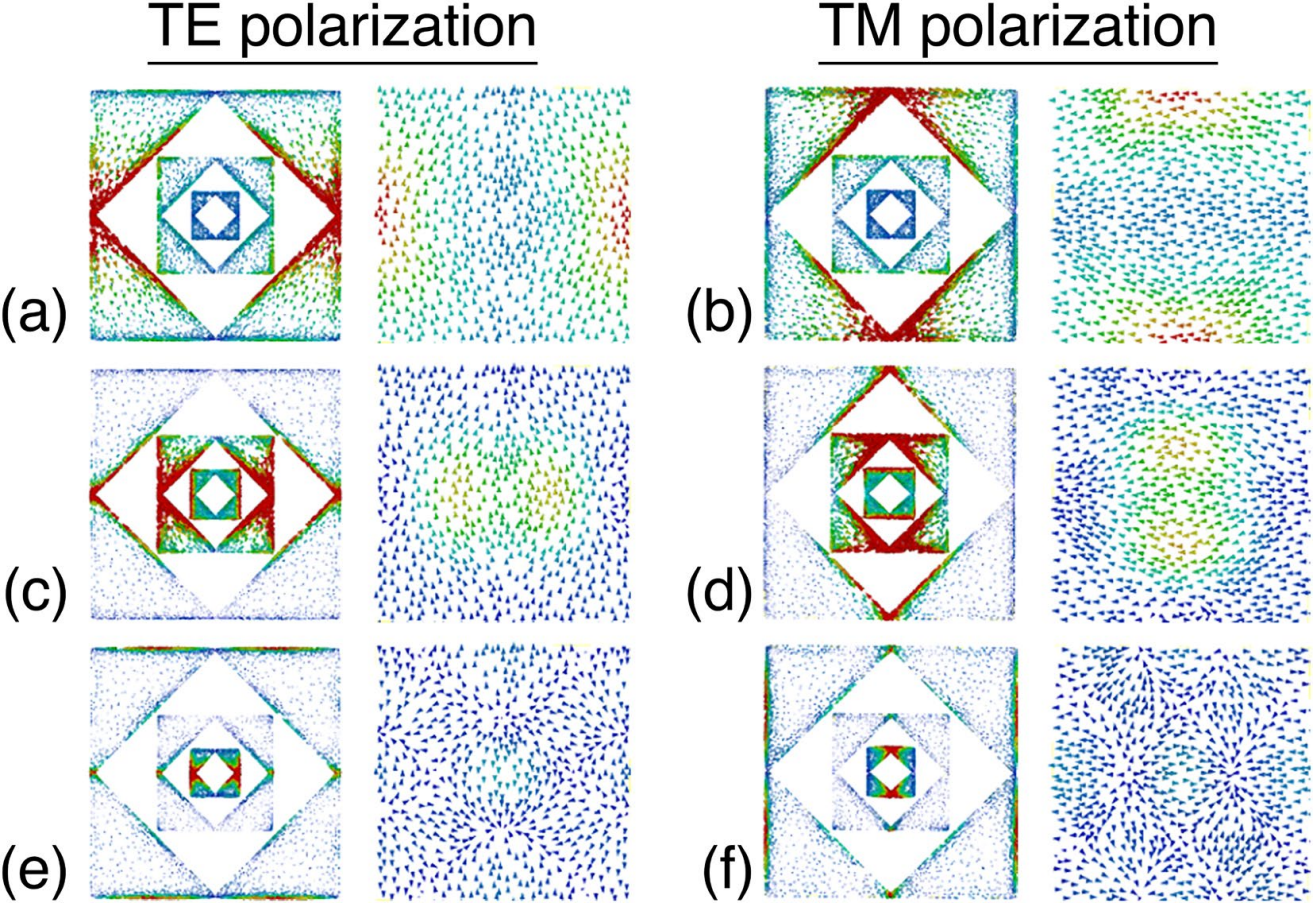
Surface current distributions on the top and bottom layer at (**a**), (**b**) 1.16THz; (**c**), (**d**) 3.41THz; (**e**), (**f**) 7.84THz for TE and TM polarization, respectively. The figure was edited using Adobe Photoshop CC 2022 (Version 23.4.1, https://www.adobe.com/products/photoshop).

**Figure 7:**
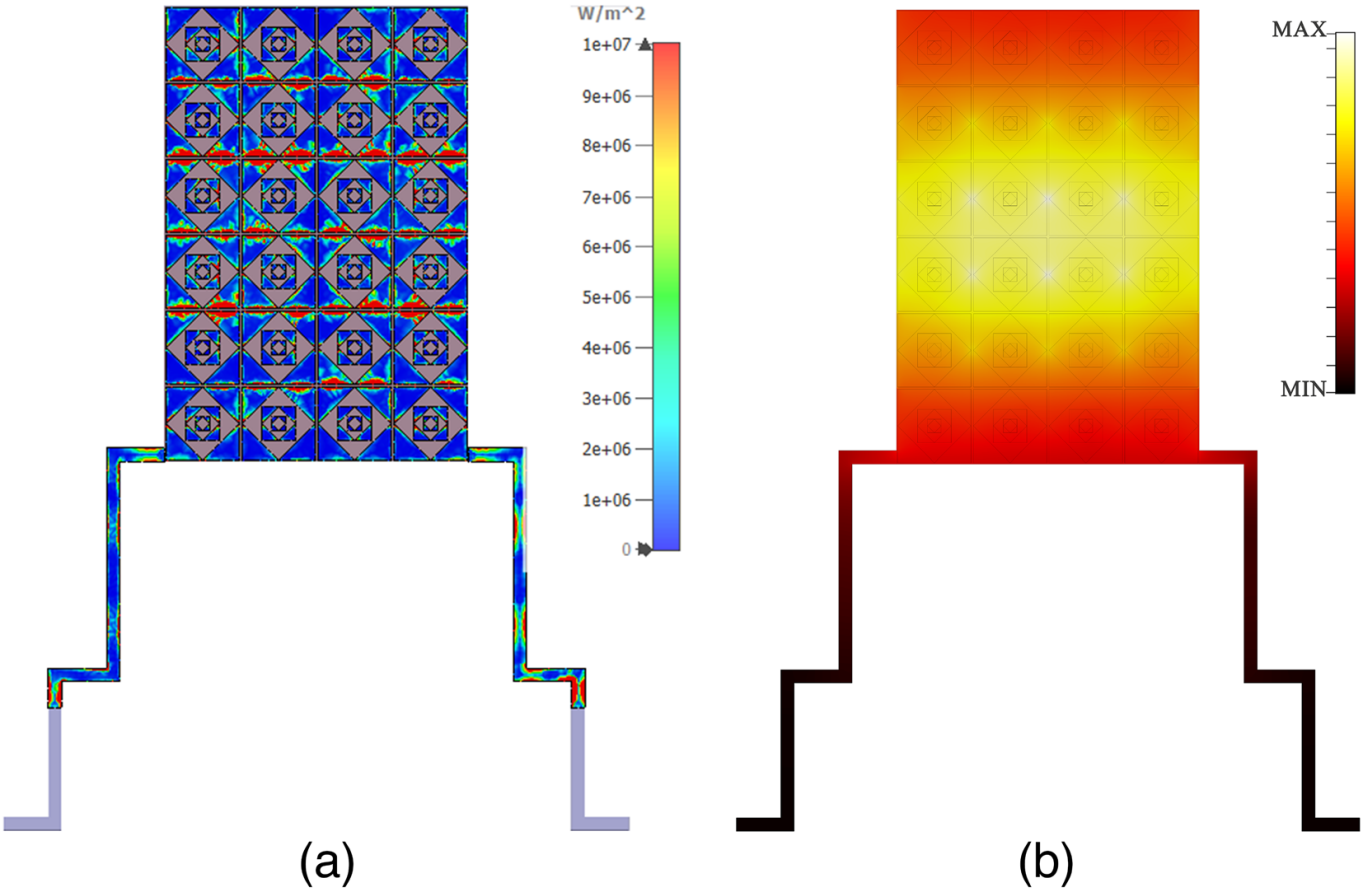
(**a**) Surface power loss density of the FMMA based focal plane array (FPA) for mode-4, (**b**) Thermal density distribution at mode-4. The figure was edited using Adobe Photoshop CC 2022 (Version 23.4.1, https://www.adobe.com/products/photoshop).

Integrating metamaterials with microelectromechanical systems (MEMS) thermal detectors is an efficient way to achieve successful sensing and imaging in the selected frequency region. Tao et al. reported a metamaterial enhanced microcantilever-based uncooled detector for microwave and THz band detection, operating at 95 GHz and 693 GHz, respectively^13^. Another approach based on bimaterial sensor probe integrated with metamaterial absorber pixels operating at 3.8 THz was experimentally demonstrated by Alves et al.^15^. The absorption spectra can be tuned statically by controlling the thickness of the dielectric layer and the dimensions of the microcantilever pixels that can enhance the sensitivity^16^. Plasmonic waves can also be excited in metamaterials to maximize the sensing and control the operation band^17^. Furthermore, active operation of THz absorption can be done using the electrical interconnection in the bimaterial microcantilever design. Xu et al. proposed SiNx based active pixels using Ti heating resistors which host the applied current to increase the temperature and bend the bimaterial microcantilever with the modulation depth of 28.1% at 0.69 THz^18^. Bending bimaterial cantilever with heat transfer during electromagnetic absorption originates the captured image signal in imaging. Another method to dynamically manipulate the absorption spectra is to apply an electrostatic field that actuates the MEMS cantilever^19^. Moreover, in line with standardization studies, Carranza et al. showed standard 180 nm complementary metal-oxide-semiconductor (CMOS) compatible metamaterial coupled with VOx and Si pn diode for security imaging devices^20^. In all these previous studies, despite the system complexity, the sensing pixels generally use single and narrowband metamaterial response which limits the sensing capacity^15,18,21–23^.

To overcome the current limitations, in this paper, we propose a novel design of a penta-band metamaterial absorber that MEMS thermomechanical bimaterial microcantilevers can actuate. The unit cell consists of triangle sectional fractal metasurfaces symmetrically positioned towards the structure center. The proposed unit cell shows penta-band absorption response at 1.1 THz, 3.4 THz, 4.9 THz, 5.9 THz, and 7.8 THz, respectively. Moreover, we also demonstrate a conceptual multiband pixel design for bimaterial microcantilever focal plane arrays (FPA). Instead of conventional single band FPA based sensing, multiple band operation can enhance the sensitivity and overall accuracy of the FPA array systems. By combining fractal sections resonating at different frequencies, the proposed metamaterial absorber can deflect the bimaterial microcantilevers at multiple bands. The proposed fractal design procedure of the multiband absorber can be used for further investigations of MEMS-based real-time uncooled imaging systems.

## Design

### Fractal metamaterial absorber (FMMA) and sensing pixel

We called the proposed unit cell fractal metamaterial absorber (FMMA). The design principle of the FMMA is based on the coupling of multiple resonant frequencies. Self-similar fractal resonators in one unit cell can provide multiple band spectral characteristics. The proposed unit cell satisfies the superposition principle, and unify the absorption response of each resonator. Fig.1a presents the 3D perspective view of FMMA. Fig. 1b,c show the top view and the side view of the FMMA. The FMMA unit cell is in the single-layer form of the fractal array. The fractal array consists of three resonators. Each resonator comprises four identical triangular-like sections with a centered square gap rotated $45^{\circ }$ on the x-y plane. The scale factor of the square gap is $1/\sqrt{2}$.

We derived the following iterative formula to calculate the dimensions of the resonator arrays.

1 $$\begin{aligned} w_{n+1}=0.5w_n-g\sqrt{2} \end{aligned}$$

where n is the number of the total resonator and $w_n$ is the corresponding side length of the $n^{th}$ resonator. The number n is an integer between {1,2} since the FMMA has three fractal resonators. The side lengths of the fractal resonators are $w_1=33$ $\mu$m, $w_2=15.08$ $\mu$m, and $w_3=6.13$ $\mu$m, respectively. The overall side length of the unit cell is $w_s=34$ $\mu$m. The gap distance g is 1 $\mu$m.

The proposed FMMA unit cell is compatible with micro-electro-mechanical systems (MEMS)-based fabrication. The dielectric material is the silicon dioxide ( $SiO_2$ ) with a dielectric constant of 3.99 and a loss tangent of 0.025^16,24^. $SiO_2$ shows stable dielectric properties during the high-energy photon absorption which makes it an efficient oxide material. The thickness of $SiO_2$ layer is $h_d=4$ $\mu$m. The top fractal pattern is aluminum (*Al*). The back of the dielectric layer is covered with *Al* ground plane with an electric conductivity of $1.0x10^7$ S/m and thickness of $h_d=0.1$ $\mu$m. The proposed unit cell has a $34$ $x$ $34$ $\mu m^2$ surface.

The proposed fractal metamaterial absorber can be used as a sensing platform for real time optomechanical uncooled imaging^21,23,25,26^. We present an FMMA based sensing pixel design for bimaterial microcantilever focal plane array (FPA) imaging systems. The bimaterial effect is the origin of FPA based imaging. Fig.2a shows the 3D view of the proposed single pixel in the FPA. Each pixel consists of an FMMA array containing one $4$ $x$ $6$ rectangular unit cell matrix, and two symmetrical bimaterial microcantilevers anchored on the glass substrate. The overall surface area of the FMMA array is 144*x*216 $\mu m^2$.

The bimaterial microcantilever is made of $SiO_2$ and *Al*, segmented into one deformation leg and one thermal isolation leg. Among the standard MEMS materials, $SiO_2$-*Al* combination gives the optimum sensitivity^25^. The bimaterial legs are integrated to the freestanding FMMA. When THz radiation is incident on the focal plan array pixels, the FMMA arrays convert the incident radiation to heat and transfer the heat to the bimaterial cantilevers. The heat deflects the bimaterial legs due to the mismatch of the thermal expansion coefficients. Fig. 2b illustrates the thermomechanical deflection of the bimaterial sensor. $L_B$ is the length of the bimaterial leg. $\Delta h$ and $\Delta \theta$ represent the linear displacement and angular deflection of the absorber, respectively. The linear displacement $\Delta h$ is ignored when $\Delta h \ll L_B$. In this case, the thermomechanical sensitivity of the FMMA sensing pixel is defined as the angular displacement per unit temperature change as follows^22,26^:

2 $$\begin{aligned} S_T=\frac{d\theta }{dT}=\frac{L_B^2(t_1+t_2)3(\alpha _1-\alpha _2)}{t_2^2Y_E} \end{aligned}$$

where

3 $$\begin{aligned} Y_E=\left[ 4+6\frac{t_1}{t_2}+4\frac{t_1^2}{t_2^2}+\frac{E_1}{E_2}\frac{t_1^3}{t_2^3}+\frac{E_2}{E_1}\frac{t_2}{t_1}\right] \end{aligned}$$

t, $\alpha$ and E represent the thickness, thermal expansion coefficient and Young’s modulus of the corresponding layer in the bimaterial leg, respectively. The most crucial parameter of bimaterial detector is the responsivity, which is given by:

4 $$\begin{aligned} R=\frac{A S_t}{G_t}1/\sqrt{1+\omega ^2\tau ^2} \end{aligned}$$

where A is the absorptivity of the FMMA array, $\omega$ is the angular frequency of the incident THz signal, $\tau$ is the response time and $G_t$ is thermal conductance of the array. It is worthy to note that high absorption, high thermomechanical sensitivity, and low thermal conductance are required to maximize the responsivity of the bimaterial detector. During the operation, the angular deflection is readout by an optical readout system.

We used CST Microwave Studio 3D full-wave solver for the electromagnetic characterization^27^. The total absorptivity of the designed structure can be determined out by using $A(\omega )$ = 1 - $T(\omega )$ - $R(\omega )$. $T(\omega )$ and $R(\omega )$ represent the transmissivity and reflectivity, respectively. This equation can be modified as $A(\omega )$ = 1 - $\vert {S_{11}(\omega )}\vert ^2$ - $\vert {S_{21}(\omega )}\vert ^2$, where $S_{11}(\omega )$ and $S_{21}(\omega )$ reflection and transmission coefficient, respectively. The bottom ground acts as a perfect reflector layer, and satisfies the skin depth condition during the operation, enabling zero THz signal transmission, $S_{21}(\omega )=0$ . Hence, the total absorptivity is reduced to the following straightforward formula of $A(\omega )$ = 1 - $\vert {S_{11}(\omega )}\vert ^2$. The parametric analysis is carried out to reveal the impact of the dimensions on the absorption. $w_1$, g and $h_d$ are modified by leaving other parameters constant. As $w_1$ increases, all absorption peaks shift towards the lower frequencies. Moreover, while the absorptivity levels look stable at mode-1 and mode-2, it slightly varies with $w_1$ for the other modes. To achieve maximum absorption in all bands, $w_1$ is optimized to 33 $\mu$m on which the other fractal dimensions directly depend as seen in (1). The gap parameter g is varied from 0.6 $\mu$m to 1.4 $\mu$m. g can be set to 0.86 $\mu$m to get individual broadband absorption with a bandwidth up to 0.5 THz at mode-5. To obtain penta-band response of the FMMA g is optimized to 1 $\mu$m. The $SiO_2$ thickness $h_d$ is also swept from 2 $\mu$m to 6 $\mu$m, and optimized to 4 $\mu$m in order to maximize the dielectric loss.

## Results and discussions

When the effective impedance of the FMMA is matched to free space impedance, the perfect absorption is achieved. The effective impedance of the FMMA can be evaluated using the following equation.

5 $$\begin{aligned} Z_{FMMA}(\omega )=\frac{1+S_{11}(\omega )}{1-S_{11}(\omega )} \end{aligned}$$

Figure 3a plots the normalized effective impedance of the FMMA with frequency. The custom computer code was executed to calculate the normalized impedance based on the reflection. As shown in Fig. 3a, at absorption peaks, the real part of the FMMA impedance is close to 1, and the imaginary part is near to 0. Fig. 3a also shows the reflection spectra. The fractal unit cell poses 5 reflection dips, i.e. zero reflection, at the absorption bands where the perfect impedance matching is achieved^28^. Figure 3b shows the absorptivity of FMMA for TE (transverse electric) and TM (transverse magnetic) polarizations under normal THz wave illumination. The fractal unit cell shows five absorption peaks A1=99.63%, A2=99.97%, A3=98.44%, A4=99.73%, and A5=95.88% corresponding to five resonance bands 1.16 THz (mode-1), 3.41 THz (mode-2), 4.93 THz (mode-3), 5.98 THz (mode-4), and 7.84 THz (mode-5), respectively. The each mode provides bandwidths of 35.5 GHz, 114.0 GHz, 131.9 GHz, 146.1 GHz, and 264.2 GHz, respectively. The corresponding bandwidth (BW) ratio can be determined as follows:

6 $$\begin{aligned} BW ratio=\frac{f_{max}-f_{min}}{f_c} \% \end{aligned}$$

where $f_{max}$ and $f_{min}$ represent the upper and lower threshold frequencies corresponding to 90% absorption level, and $f_c$ is the related center frequency^29^. According to Eq. 6, BW ratio is 3.1%, 3.34%, 2.67%, 2.44% ,and 3.36% from mode-1 to mode-5.

Each individual fractal resonator is further analyzed to better understand the multiband absorption mechanism of the FMMA. Fig. 3c compares the absorptivity curves obtained for each resonator and the proposed FMMA. The resonator-1 (S1) excites 3 absorption modes (black curve), namely, mode-1, mode-3, and mode-4, respectively. Similarly, while the resonator-2 (S2) excites mode-2 and mode-5 (red curve), the resonator-3 (S3) forms mode-6 and mode-7 (blue curve). Figure 3c proves that the proposed FMMA satisfies the principle of superposition, and provides 5 absorption peaks as the linear combination of 7 peaks corresponding to the resonators(orange curve). It is interesting to note that the mode-5 in the proposed FMMA response extends its bandwidth to 264.2 GHz covering the mode-6 and mode-7.

The incident THz radiation bend the sensor plane out of the substrate plane. Therefore the incident angle dependent spectral response is one of the most critical parameters in absorber design. First the spectral response with different polarization angles is investigated. Figure 4a,b shows absorption curves for increased polarization angles ($\phi$) up to 180 degrees under normal THz wave illumination. It is observed that the proposed absorber indicates polarization-insensitivity due to structural symmetry on the unit cell plane. The absorption performance of the designed FMMA with oblique THz wave incident is also carried out for TE and TM polarization modes. For TE polarization as presented in Fig. 4c, the FMMA provides near perfect absorption for the incident angles up to $60^{\circ }$ at 1.16 THz and 3,41 THz. At the band of mode-3, 4.93 THz, the FMMA is perfectly active up to $45^{\circ }$. At the mode-4, although the absorption region slightly shifts to lower bands, FMMA provides absorptivity higher than 80% up to $45^{\circ }$. It is interesting to notice that at mode-5, 7.84 THz, the absorptivity decreases for higher angles of incidence due to the effect of the high-order diffraction as indicated with region-A for TE mode. Moreover, the mode-5 absorption is split to two regions. The high-order diffraction problem can be overcome by matching the side of the unit cell to subwavelength dimension^30^. Similar absorption characteristics are observed at all modes for TM polarization as shown in Fig. 4d.

### Constitutive parameters of the FMMA

The resonance mechanism of the proposed FMMA can be explained by evaluating the constitutive electromagnetic parameters. The effective electric permittivity $\varepsilon _{eff}(\omega )$ and the magnetic permeability $\mu _{eff} (\omega )$ is calculated to reveal that how the proposed absorber interacts with the incident electric and magnetic fields. The effective constitutive parameters $\varepsilon _{eff}(\omega )$ and $\mu _{eff} (\omega )$ are given by^31,32^

7 $$\begin{aligned} \varepsilon _{eff}(\omega )\cong & {} X \frac{1-(S_{21}(\omega )-S_{11}(\omega ))}{1+(S_{21}(\omega )-S_{11}(\omega ))} \end{aligned}$$

8 $$\begin{aligned} \mu _{eff}(\omega )\cong & {} Y \frac{1-(S_{21}(\omega )+S_{11}(\omega ))}{1+(S_{21}(\omega )+S_{11}(\omega ))} \end{aligned}$$

where $$X=Y=2/jk_od$$.

Figure 5 compares the real and imaginary parts of the effective permittivity and the permeability. It is clearly seen that dramatic changes are observed in permittivity and permeability around the mode regions. Particularly, imaginary part of the magnetic permeability shows abrupt changes at the frequencies corresponding to the absorption modes. This outcome proves that FMMA has the dominant magnetic resonance characteristics.

To explain the absorption mechanism, the surface current distribution analysis is performed for all operation modes. Figure 6 shows how the TE and TM polarized vector current densities of the resonance modes behave in the S1, S2 and S3 resonators. As shown in Fig. 6a,b, the current density at 1.16 THz (mode-1) is mainly provoked by the resonator S1. It is observed that this interaction is concentrated in the left/right and top/bottom central regions of the R1 for TE and TM polarizations, respectively. Similar characteristics is also active for the mode-3 and mode-4 of the S1. Furthermore, the resonator R2 generates a large magnitude of current at 3.41 THz (mode-2), as shown in Fig. 6c,d. The incident THz wave dominantly couples to the resonator S3 at 7.84 THz, and creates mode-5 localized around the center of unit cell. It is apparent that the resonators S1, S2 and S3 in different dimensions produce different modes, which explains the origin of the penta-band response. On the other hand, at all modes of the FMMA, the current flow in the bottom surface is anti-parallel (out of phase) to that of the top surface. This type of vortex current behavior excites a dense magnetic resonance in response to the incident wave. Therefore, the resonance mechanism of the FMMA is mainly based on the magnetic interaction.

The proposed absorber generates a corner mode transversely at the central areas as shown in Fig. 6. However, when the polarization angle varies, all vertical and horizontal corners become active for absorption. It is worth to note that the corners are the active absorption regions having geometric discontinuities where the light-matter interaction is strongly provoked. Therefore, the structure maintains the same absorptivity due to the collective response of all active corners in response to the incident THz wave with different polarization angles. It proves that the proposed structure shows nearly polarization-insensitive even it has 90 degree rotation symmetry. The proposed FMMA based FPA pixel is also investigated in the simulation environment. Figure 7a shows the surface power loss density distribution during the operation. It is clearly seen that the most of the energy is dissipated around the metallic edges. We attribute this behavior that the incident energy is mainly lost at the regions where the high light-matter interaction is observed. Furthermore, Fig. 7b demonstrates the normalized thermal heat distribution in response to incident THz energy. As shown, the thermal heat response of PFA pixel is localized at the array center.

## Conclusions

In conclusion, a penta-band fractal metamaterial absorber (FMMA) is numerically investigated and presented. The proposed FMMA structure shows five near perfect absorption modes based on the systematically combined of three fractal interwoven resonators. All absorption modes provide sufficient bandwidth within the operation band covering 1.1-7.8 THz region. Based on the multimode characteristics, we propose the concept model of a FMMA based pixel structure for bimaterial microcantilever FPA sensing. The surface area of the FMMA pixel is 144*x*216 $\mu m^2$. Under THz illumination, the mechanical deformation of the FMMA pixels can be conveniently readout by optical methods without any on-chip design. The proposed FMMA is polarization-independent, and poses near perfect absorption for the first four mode over a wide incident angle range up to $45^{\circ }$ in case of TE and TM modes, respectively. The fractal design collectively hosts large vortex surface currents in response to the incident THz waves, therefore the magnetic resonation dominantly governs the absorption. The proposed fractal absorber design procedure can be used for further investigations of bimaterial microcantilever FPA sensing technologies suffering from low sensitivity, especially in the security and medical fields.

## Methods

### Numerical simulation

The simulations are carried out using the 3D full-wave electromagnetic solver CST Microwave Studio 2019. To obtain the figure-of-merits including S-parameters, absorptivity, vector current distributions, field profiles, etc., the simulation setup settings are as follows: Frequency domain solver is used. The second order solver is chosen, which provides good accuracy. The accuracy is set to 10^–4^. The tetrahedral mesh type is employed. The average number of tetrahedron is around 50000. The number of result data samples is set to 1001. The unit cell boundary condition with Floquet-port is applied, which introduces periodic boundary condition in the x- and y-axis, and open boundary condition along the z-axis. Furthermore, effective medium theory is used to verify the simulation outcomes. A custom code is executed in MATLAB in order to calculate the effective permittivity and permeability of the proposed metamaterial structure. These constitutive electromagnetic (EM) parameters are extracted by using the magnitude and the phase information of S-parameters. Therefore the distance to the reference plane in simulation environment is set to -λ/4 to prevent the phase error in effective EM parameter calculation.

## Data Availability

The datasets used and/or analysed during the current study available from the corresponding author on reasonable request.
